# Identification of Sialic Acid Linkages on Intact Glycopeptides via Differential Chemical Modification Using IntactGIG-HILIC

**DOI:** 10.1007/s13361-018-1931-0

**Published:** 2018-04-12

**Authors:** Shuang Yang, Wells W. Wu, Rong-Fong Shen, Marshall Bern, John Cipollo

**Affiliations:** 1 Laboratory of Bacterial Polysaccharides, Division of Bacterial, Parasitic and Allergenic Products, Center for Biologics Evaluation and Research, Food and Drug Administration, G614, Bldg 75, 10903 New Hampshire Ave, Silver Spring, MD 20993 USA; 2 Facility for Biotechnology Resources, Center for Biologics Evaluation and Research, Food and Drug Administration, Silver Spring, MD 20993 USA; 3 Protein Metrics Inc., 1622 San Carlos Ave, Suite C, San Carlos, CA 94070 USA; 4 Laboratory of Bacterial Polysaccharides, Division of Bacterial, Parasitic and Allergenic Products, Center for Biologics Evaluation and Research, Food and Drug Administration, G637, Bldg 52/72, 10903 New Hampshire Ave, Silver Spring, MD 20993 USA

**Keywords:** Sialoglycopeptide, NeuAc2,3, NeuAc2,6, Amidation, Esterification, HILIC

## Abstract

Mass spectrometric analysis of intact glycopeptides can reveal detailed information about glycosite, glycan structural features, and their heterogeneity. Sialyl glycopeptides can be positively, negatively, or neutrally charged depending on pH of their buffer solution and ionization conditions. To detect sialoglycopeptides, a negative-ion mode mass spectrometry may be applied with a minimal loss of sialic acids, although the positively charged or neutral glycopeptides may be excluded. Alternatively, the sialyl glycopeptides can be identified using positive-ion mode analysis by doping a high concentration of sodium salts to the analytes. Although manipulation of unmodified sialoglycopeptides can be useful for analysis of samples, less than optimal ionization, facile loss of sialyl and unfavorable ionization of accompanying non-sialyl peptides make such strategies suboptimal. Currently available chemical derivatization methods, while stabilizing for sialic acid, mask sialic acid linkage configuration. Here, we report the development of a novel approach to neutralize sialic acids via sequentially chemical modification that also reveals their linkage configuration, often an important determinant in biological function. This method utilizes several components to facilitate glycopeptide identification. These include the following: solid phase derivatization, enhanced ionization of sialoglycopeptides, differentiation of sialic acid linkage, and enrichment of the modified glycopeptides by hydrophilic interaction liquid chromatography. This technology can be used as a tool for quantitative analysis of protein sialylation in diseases with determination of sialic acid linkage configuration.

**Graphical Abstract:**
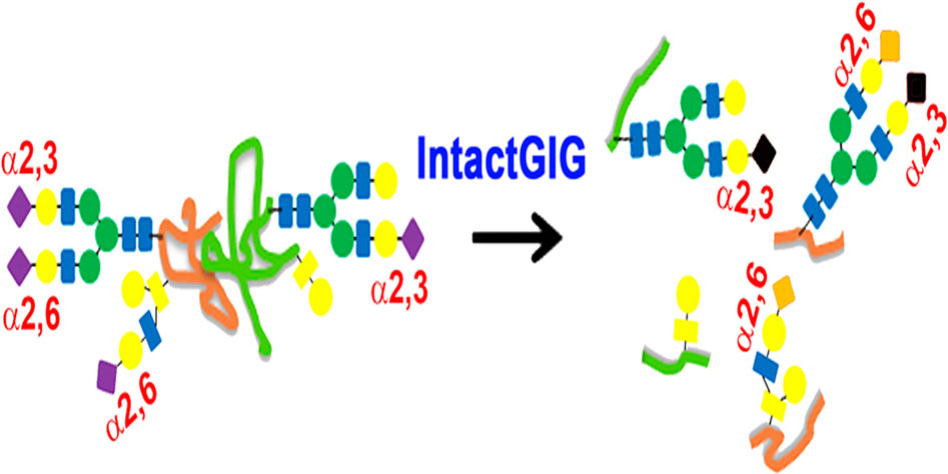
ᅟ

ᅟ

## Introduction

Glycosylation is one of the most abundant post-translational modifications and is often found on secreted, membrane, or extracellular matrix proteins. These modifications can serve as signature patterns that act as markers for the physiological state of a living organism indicating a normal or pathological state [1]. Beyond acting as a simple marker, a change in glycosylation status may impact protein function, folding [2], stability [3, 4], and modulatory effects on cell surface receptor interactions [5]. Aberrant glycosylation is associated with many disease states and thus development of methods that can reveal important features of these chemical entities is desirable.

As a terminal substitution on many glycans, sialic acid plays a critical role in a range of recognition events in biology. For instance, avian influenza hemagglutinin (HA) primarily recognizes sialylα2,3 residues while human influenza favors sialylα2,6 substitutions and swine influenza recognizes both [6, 7]. Pandemics can arise when a mutation in avian or swine influenza HA shifts sialyl specificity toward sialylα2,6. A method, such as the one presented here, that can detect these subtle differences, α2,3 versus α2,6-linked sialic acids, at the glycosylation site of the resident protein can provide an important tool to better understand the role of sialic acids in such biological processes.

Analysis of protein glycosylation primarily includes identification of (1) glycosite, (2) glycopeptide sequence, and (3) resident glycans. A range of methods have been developed to investigate these compounds; however, some drawbacks remain. Glycosylation sites can be determined by methods that sequentially digest samples with trypsin and PNGase. The PNGase release of glycans can be done in the presence of isotope-coded heavy water (H_2_O^18^), leading to deamidation of asparagine (N) to aspartic acid (D) labeled by O^18^ [8]. While these methods can be used successfully to identify glycosylation site and peptide sequence, glycan moiety information, specific to the glycosite, is lost. While the glycans can be collected and analyzed separately, site specific information is not revealed and the three-component picture is not complete. Other methods center on glycopeptide capture. Because each glycopeptide carries at least one glycan with vicinal hydroxyls, the glycan moiety can be oxidized chemically (e.g., sodium periodate) [9]. The oxidized glycopeptides are selectively isolated from non-glycopeptides via hydrazide chemistry and solid phase capture, followed by enzymatic release of the formerly N-linked glycopeptides [10]. However, these strategies again do not yield glycan information. They only indicate if a glycan was present via mass shift through conversion of N to D.

Released glycans can be analyzed using a range of strategies. N-glycans are typically released enzymatically whereas O-glycans are typically cleaved chemically [9, 11]. Glycans containing sialic acids require additional considerations. These sugars are often present on cell surfaces and body fluids. In addition to the influenza example provided previously, sialylation plays diverse and crucial roles in biological functions and disease states [12]. For example, increasing the sialylation of therapeutic glycoproteins can benefit their half-life in circulation [4, 13, 14]. However, their analysis can be confounded by sialic acid’s labile nature and, therefore, methods have been developed to stabilize these acidic monosaccharide components [15–17]. Using mass spectrometry, analysis of native forms can be facilitated through use of negative-ion mode, which, through ionization via loss of acidic carbonyl hydrogen, allows preservation of the sialic acid linkage [13, 15]. This can be accomplished in both MALDI (matrix-assisted laser desorption/ionization) or ESI (electrospray)-sourced instruments [18]. However, neutrally and positively charged glycans may not be representatively identified. Other strategies protect sialic acids through chemical derivatization including amidation [19], esterification [20], perbenzoylation [21], or permethylation [22]. These methods are widely used in identification of sialylated glycans in biological and clinical studies. Still, these methods do not reveal the site of glycosylation.

Analogous to methods used for detached sialoglycan analysis, negative-mode analysis has been used for enhanced detection and identification of intact sialoglycopeptides that are heavily sialylated [23]. However, glycopeptides that are not easily ionized in a negative polarity mode can be underrepresented and therefore such samples may require additional analysis in positive-ion mode. The negatively charged sialoglycopeptides may be detected in positive-ion mode through doping the running solvent with a high concentration of sodium salt, such as 0.6-mM sodium hydroxide to the mildly acidic-buffered aqueous part of the mobile phase as previously reported [24]. A caveat is ion-pairing between glycopeptides and stationary phase may be generated only for positively or negatively charged species. Thus, some glycopeptides may be selectively captured via ion-pairing interactions in liquid-chromatography (LC)-MS whereas others are poorly detected [16].

Glycopeptides can be analyzed as positively or negatively charged depending on the buffer system used. For example, peptide components arginine (R), histidine (H), and lysine (K) can be positively charged in protonated buffer whereas aspartic acid (D), glutamic acid (E), and sialic acid are negatively charged in basic buffer. Positive-ion mode is widely used for ionization of glycopeptides. However, positive-ion mode and acidic buffer are detrimental to sialoglycopeptides in that sialic acids can be partially or completely lost during sample preparation and ionization [25, 26]. Our recent work found that derivatization can protect sialic acids and improve ionization of sialoglycopeptides in MALDI and ESI [27–29]. In these strategies, the derivatized glycoproteins were directly digested from resin for glycopeptide analysis.

Derivatization of sialoglycopeptide can be an alternative to counteract charge effect by sialic acids. This approach can be designed to not only negate negative charges carried by sialic acids but also stabilize sialic acids during sample processing and ionization [25]. For example, esterification can stabilize sialoglycopeptide and such strategies have been applied for semi-quantitative determination of sialylation in antibodies [30]. Determination of specific sialic acid linkage on glycopeptides has been demonstrated using MALDI-ToF-MS after liquid phase chemical modification. Using this strategy, α2,3 forms lactone and α2,6 generates ester [31, 32]. However, it was found that the α2,3 associated lactone was unstable, quickly hydrolyzing to its carboxylic acid form [33]. Moreover, modification of sialoglycopeptide in solution faces several challenges, for instance, removal of chemical compounds after reaction and sample loss after cleanup. Therefore, it becomes apparent that performance of these derivatization steps in solid-phase would be advantageous likely providing efficient, cost-effective, and fast approach for analysis of intact glycopeptides.

Our previous work showed that derivatization of α2,6 and α2,3-linked sialic acids is implemented on solid-phase for analysis of glycans. In this study, we developed a two-step solid-phase matrix-based method for sequential derivatization of glycopeptides containing α2,6 and α2,3 (IntactGIG: intact glycoprotein immobilization for glycopeptide analysis) for analysis of intact glycopeptides. We identified ethylene diamine (EDA) for derivatization of α2,3-linked sialic acids, which can not only differentiate α2,6 and α2,3-linked glycopeptides but also prevail hydrophilicity of sialoglycopeptides after derivatization. Glycoproteins are first immobilized on the solid support, allowing for chemical reaction of sialic acids, D, E, and C-terminal. Excess reagents are added for complete chemical reaction. Reagents are removed by extensive washing steps after each reaction. Glycopeptides and peptides are directly digested from the solid support, followed by hydrophilic liquid interaction chromatography (HILIC) enrichment for mass spectrometry analysis. Glycopeptides are identified including anomeric configuration of sialic acids.

## Methods

### Materials and Reagents

All chemicals were purchased from Sigma-Aldrich unless otherwise noted. Snap-cap spin columns (SCSC), Aminolink resin, graphitized-active carbon column, HPLC-grade water, and acetonitrile (ACN) were purchased from Fisher Scientific. Ethylenediamine (EDA), p-toluidine (pT), 1-hydroxybenzotriazole hydrate (HBot), *N*-(3-dimethylaminopropyl)-*N*′-ethylcarbodiimide (EDC) (liquid), EDC·HCl (powder), urea, ammonium bicarbonate, dithiothreitol (DTT), iodoacetamide (IAA), 2,5-dihydroxybenzoic acid (DHB), *N*,*N′*-dimethylaniline (DMA), formic acid (FA), DMSO, hydrochloric acid (HCl), maltoheptaose (DP7), phosphate-buffered saline (PBS, 1×), sodium cyanoborohydride (NaCNBH_3_), sodium carbonate, sodium citrate, Tris-HCl, and trifluoracetic acid (TFA) were from Sigma-Aldrich. NaCl (5 M) was from ChemCruz Biochemicals. Peptide-*N*-glycosidase F (PNGase F), denaturing buffer (10×; 400-mM DTT and 5% sodium dodecyl sulfate), and reaction buffer (G7; 10×; 500-mM sodium phosphate, pH 7.5) were purchased from New England BioLabs (Ipswich, MA). Sequencing-grade modified trypsin was purchased from Promega Corporation (Madison, WI). HILIC SPE chromatography was prepared as follows: add Empty SPE (solid-phase extraction) frits to Grace Alltech Extract-Clean empty reservoir (1.5 mL; Fisher Scientific), load 500-μL TSKgel Amide-80 (Sigma-Aldrich), and cap resin using Empty SPE frits.

### Protein Conjugation and Modification

**Figure 1:**
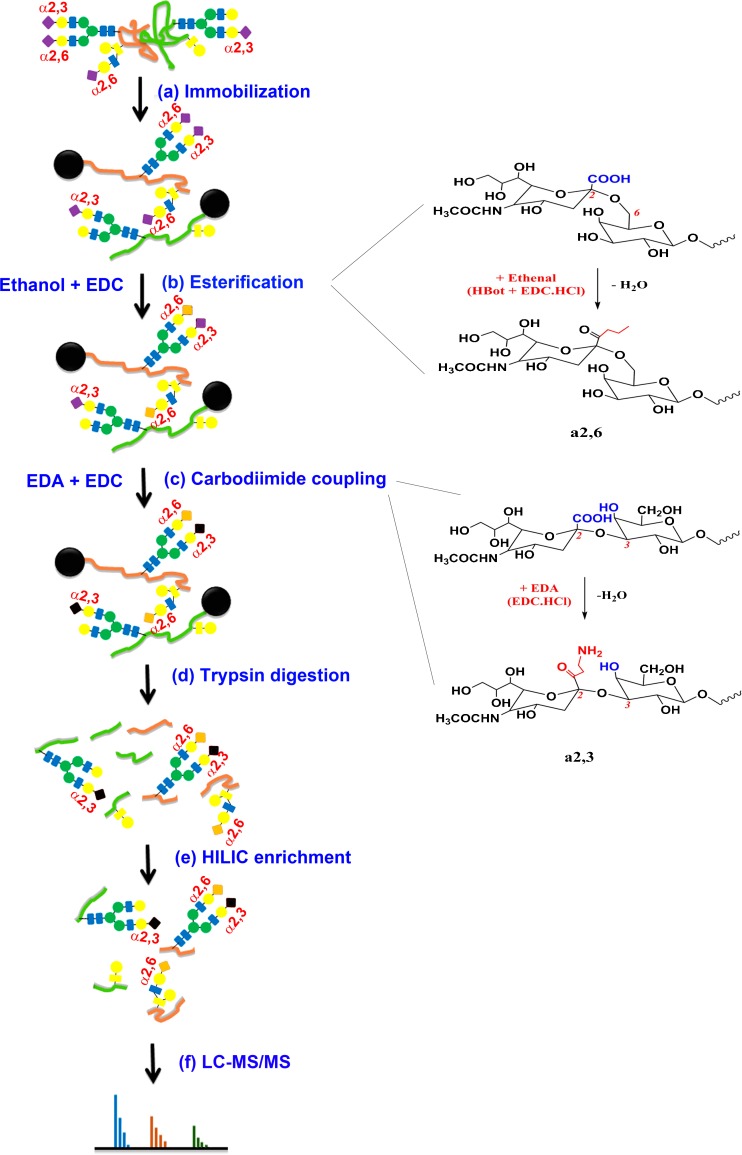
Schematic scheme of glycoprotein modification using a chemoenzymatic solid-phase method. (**a**) Proteins were conjugated to the solid support (Aminolink resin) via reductive amidation. (**b**) α2,6-Linked sialic acid, D, E, and C-terminal are derivatized by ethanol in presence of EDC and HBot. (**c**) α2,6-Linked sialic acid and remaining unmodified D, E, and C-terminal are further modified by ethylenediamine (EDA) in presence of EDC at pH 4–6. (**d**) Proteins are digested from resin using trypsin after reduction and alkylation. (**e**) The derivatized glycopeptides are enriched by HILIC SPE chromatography, and flow-through contains non-glycopeptides. (**f**) Glycopeptides are tested by LC-MS/MS and searched by Byonic and Byologic

Binding buffer (1×) was prepared by dissolving 294-mg sodium citrate and 53-mg sodium carbonate in 10-mL HPLC water. Proteins were dissolved in 500-μL 1× binding buffer. Aminolink resin was conditioned with addition of 500-μL binding buffer and repeat once (Figure 1a). Proteins were added to pre-conditioned resin in a SCSC column and incubated for 4 h at room temperature. The reduction solution (50-mM NaCNBH_3_) was added to sample for another 4 h. The resin was then washed by 500-μL 1× PBS twice; sample was further incubated in 50-mM NaCNBH_3_ in 1× PBS for 4 h. The resin was treated by 1× Tris-HCl in the presence of 50-mM NaCNBH_3_ to block any active aldehyde sites on resin [34, 35]. Two-step chemical modifications were performed on samples for stepwise sequential derivatization of α2,3- and α2,6-linked sialic acids [33]. For esterification, samples were incubated for 2 h at 37 °C in 500-μL ethanol solution containing 0.25-M EDC·HCl and 0.25-M HBot (Figure 1b). Samples were washed by 500-μL 1-M NaCl three times, followed by DI water; for carbodiimide coupling (amidation), samples were treated with 465-μL EDA solution containing 400-μL 1-M EDA, 40-μL EDC, and 25-μL HCl (final pH at 4.0–6.0, add either EDC or HCl if necessary). The reaction was allowed to proceed for 3 h and repeated once with freshly prepared EDA solution (Figure 1c). After two-step reactions, samples were extensively washed using the following solutions: 10% TFA (500 μL, thrice), 10% ACN (500 μL, thrice), 1-M NaCl (500 μL, thrice), and HPLC water (500 μL, thrice). Samples should be kept in solution when stored at 4 °C (to prevent resin from drying).

### On-Bead Protein Digestion and HILIC Enrichment

Proteins conjugated on resin were first digested by trypsin (50:1) (Figure 1d). Prior to tryptic digestion, proteins were alkylated by 12-mM DTT in 1-M NH_4_HCO_3_ and 8-M urea at 37 °C for 1 h, followed by adding IAA to a final concentration of 16 mM (1 h at room temperature in the dark). After alkylation and reduction, samples were washed by 1-M NaCl, HPLC water, and 25-mM NH_4_HCO_3_ sequentially (twice). Trypsin digestion was performed at 37 °C overnight in 100-mM NH_4_HCO_3_ and 1-M urea. To compare performance of sialoglycopeptides by chromatographic enrichment, 50% peptides were enriched by HILIC SPE chromatography, while others were cleaned by C-18 SPE (Figure 1e). HILIC enrichment was performed as follows: pre-condition HILIC column using 0.1% TFA (1 mL, thrice) and 80% ACN in 0.1% TFA (1 mL, three times), load samples in 80% ACN/0.1% TFA (re-load flow-through once), wash column by 80% ACN (0.1% TFA) (1 mL, thrice), elute samples by 60% ACN (0.1% TFA) (1 mL, twice), 40% ACN (0.1% TFA) (1 mL, twice), and 0.1% TFA (1 mL, twice), and pool samples. The eluates were dried at Speed-Vac (Thermo Fisher) and re-suspended in 0.2% FA.

### LC-ESI-MS/MS

Peptides (1 μg) were analyzed by LC/MS/MS using a Thermo Fisher Ultimate LC and Fusion Orbitrap MS (San Jose, CA). Briefly, peptides were first loaded onto a trap cartridge (Thermo Fisher PepMap, C18, 5 μm, 0.3 × 5 mm), then eluted onto a reversed phase Easy-Spray column (Thermo Fisher PepMap, C18, 3 μm, 100 Å) using a linear 120-min gradient of ACN (2–50%) containing 0.1% FA at 250 μL/min flowrate. The eluted peptides were sprayed into the Fusion Orbitrap. The data-dependent acquisition (DDA) mode was enabled, and each FTMS MS1 scan (120,000 resolution) was followed by linear ion-trap MS2 scans using top speed (acquire as many MS2 scans as possible within 1-s cycle time). Precursor ion fragmentation took place in the HCD cell with CE energies of 33 and 27, respectively, for general peptides and glycopeptides. Automatic gain control (AGC) targets were 2.0 × 10^5^ and 1.0 × 10^4^, respectively, for MS1 and MS2. The spray voltage and ion transfer tube temperature were set at 1.8 kV and 250 °C, respectively. MS spectra were analyzed by Byonic and Byologic software (Protein Metrics, CA).

## Results and Discussion

### Mass Shift by Esterification and Amidation

Any carboxylic acid (▬COOH) may react with either ethanol during esterification or EDA during amidation. Esterification is performed first to derivatize α2,6-linked sialic acid without reaction with α2,3-linked sialic acid. Theoretically, aspartic acid (D), glutamic acid (E), and protein C-terminal may be modified by ethyl esterification during this first stage. It is possible that these amino acids may react with EDA during the second stage of derivatization. Expected mass shifts are listed in Table 1 (28.03 Da for esterification and 42.06 Da for amidation).

**Table 1:** Mass Shift of Glycopeptide Modification by Ethyl Esterification and Ethylenediamine (EDA) Amidation. Ethyl Esterification Modifies α2,6-Linked Sialic Acids, While EDA Derivatizes α2,3-Linked Sialic Acids

Group	Modification	Reagent	Mass shift (Da)
2,3-Linked sialic acid	Amidation	EDA	42.058183
2,6-Linked sialic acid	Esterification	Ethanol	28.031301
Aspartic acid (D)	Amidation	EDA	42.058183
	Esterification	Ethanol	28.031301
Glutamic acid (E)	Amidation	EDA	42.058183
	Esterification	Ethanol	28.031301
Protein C-terminal	Esterification	Ethanol	28.031301
	Amidation	EDA	42.058183

**Figure 2:**
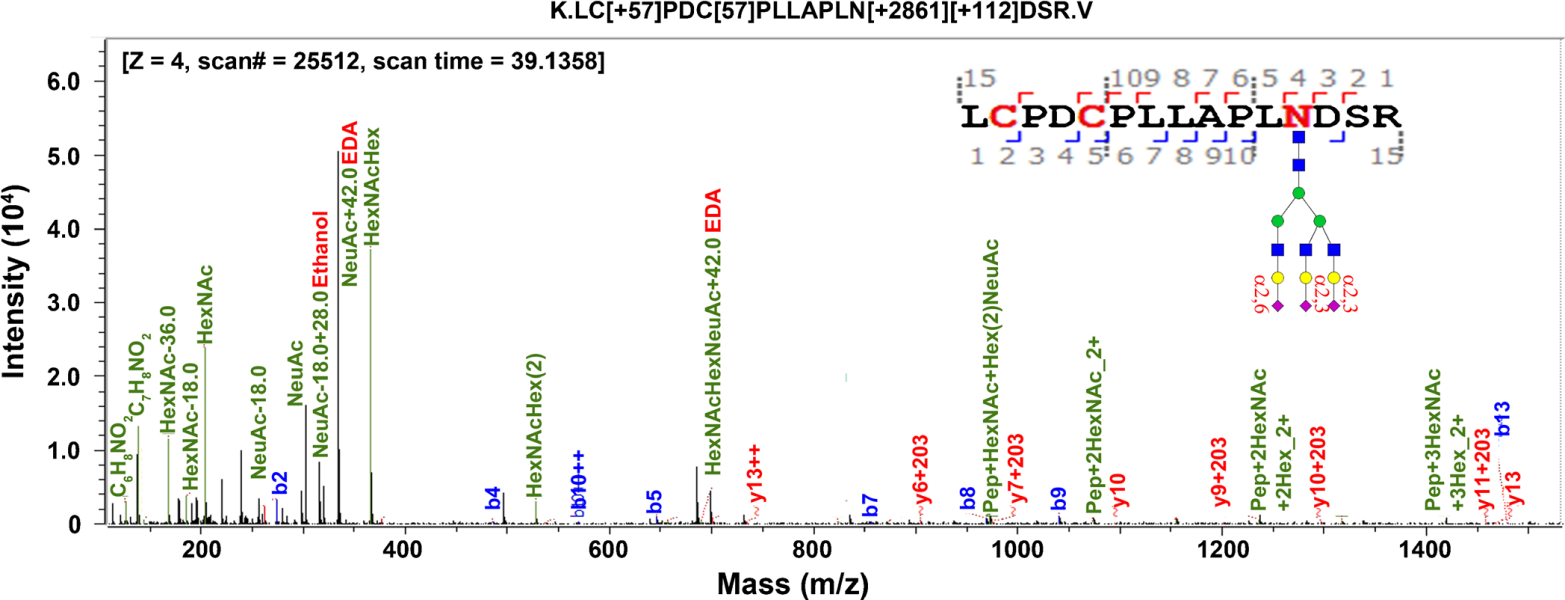
Oxonium ions of MS/MS on intact glycopeptides after sialic acid derivatization. Sialic acids are present with ethyl and/or ethylenediamine, depending on their linkages

To determine whether sialic acids are successfully derivatized by ethanol and/or EDA, we inspected fragment ions of tandem mass spectra. Oxonium ions bearing mass shifts consistent with both ethyl and EDA modified NeuAc of glycopeptide, LC[+57.0]PDC[+57.0]PLLAPL*N[+2861.1][+112.0]*DSR, were observed as shown in Figure 2. Other oxonium ions, C_6_H_8_NO_2_, C_7_H_8_NO_2_, and HexNAc, also observed, were consistent with literature reports [36]. This glycopeptide has one N-glycan, whose sialic acids have been modified by both ethanol and EDA. Their fragment peaks are present at 334.1 Da for NeuAc + EDA (α2,3-linked), 316.1 Da for NeuAc + EDA − H_2_O, and 302.1 Da for NeuAc + ethyl − H_2_O (α2,6-linked). Oxonium ions also include HexNAc − H_2_O, HexNAcHex, and HexNAcHex(2). These results verified that sialic acids are successfully modified by ethyl esterification and EDA amidation.

**Figure 3:**
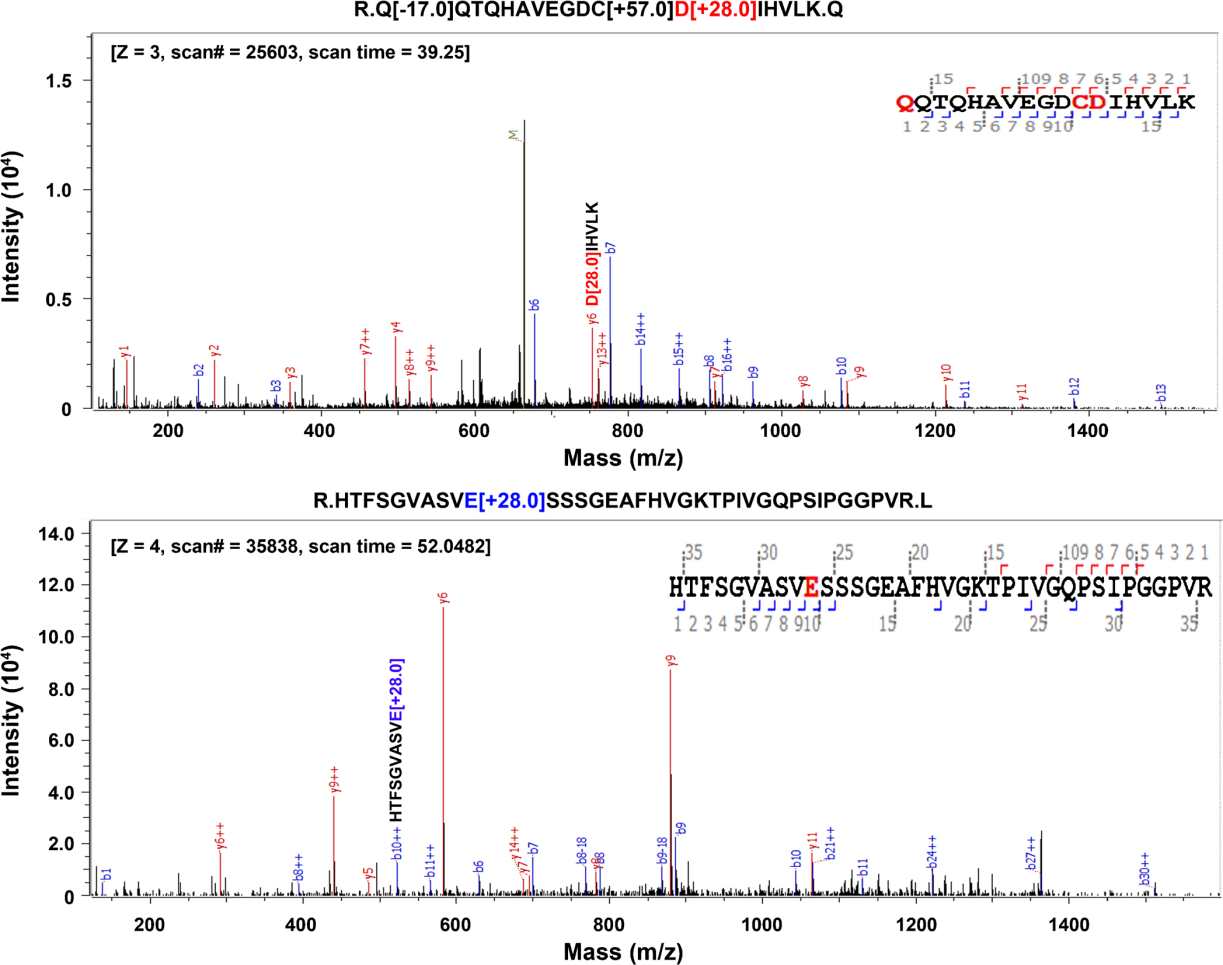
Modification of carboxylic acid on D (aspartic acid) and E (glutamic acid). Acidic amino acids, together with protein C-terminal, are derivatized by ethyl esterification. D or E are also modified by ethylenediamine (EDA) amidation

To determine if one or both derivatizations had taken place on the carboxylic acids of D, E, or C-terminal, we performed data searches using esterification only (option 1), both esterification and amidation (option 2), and amidation only (option 3) on same LC-MS experimental dataset. Fetuin from fetal bovine serum was tested. Search parameters are listed in Table 2. Results showed that some D and E are only derivatized by ethyl esterification during modification of α2,6-linked sialic acids. As shown in Figure 3, *y6* of D in peptide, QQTQHAVEGDC*D[+28.0]*IHVLK, carries an ethyl-derivatized peptide fragment. Peptide, HTFSGVASV*E[+28.0]*SSSGEAFHVGKTPIVGQPSIPGGPVR shown in the lower panel has a fragment ion (*b10*) that is also modified by ethyl esterification. Peptides can also be modified by both ethanol and EDA. Table 3 lists the relative abundance of modified D and/or E by ethanol or EDA. Likely because ethyl esterification is performed prior to EDA amidation for α2,3-linked sialic acids, D or E is primarily modified by ethanol derivatization. Table 3 lists the percentages of modification found on D and E on fetuin for the analyses. Option 2 (ethyl and EDA) delivers 27% modification. Option 1 (ethyl only) delivers 25% and option 3 (EDA only) delivers 7.6% modification. These results indicated that both modifications can occur on D or E. Thus, we use search “option 2” (Table 2) in Byonic as the input for glycopeptide analysis.

**Table 2:** Search Parameters Used in Byonic Software. Options 1, 2, and 3 Were Used to Compare the Modification of D, E, and/or Protein C-Terminal

Setting	Item	Parameter	Remark
Digestion and instrument	Missed cleavage	1	
	Precursor mass tolerance	5 ppm	
	Fragmentation	QToF & HCD	
	Fragment mass tolerance	0.3 Da	
Spectrum input	Maximum precursor mass	10 kDa	
	Maximum # of precursors per MS2	1	
Peptide output	Manual score cut	10	
	Show all *N*-glycopeptides	Yes	
Protein output	Protein FDR	2%	
Amino acid modification	Carbamidomethyl	57.021454	C, fixed
	Oxidation	15.994915	M, rare
	Gln → pyro-Glu	− 17.026549	Nterm Q, rare
	Gln → pyro-Glu	− 18.010565	Nterm E, rare
	Ammonia loss	− 17.026549	Nterm C, rare
Option 1	Ethyl esterification	28.031301	D, E, Cterm, rare
Option 2	Ethyl esterification	28.031301	D, E, Cterm, rare
	Amidation	42.058183	D, E, Cterm, rare
Option 3	Amidation	42.058183	D, E, Cterm, rare
Sialic acid modification	a2,-Linked	28.031301	
	a2,3-Linked	42.058183

**Table 3:** Relative Abundance of Modified D and E by Ethyl Esterification and EDA, Respectively

Peptide	Abundance (%)				Missed cleavage
	Unmodified	Ethyl	EDA	Total abundance in identified peptides	
CDSSPDSAEDVR	0.79	0.70	0.01	1.50	0
HTLNQIDSVK	0.39	0.00	0.01	0.40	0
LCPDCPLLAPLNDSR	15.39	3.02	1.61	20.02	0
LCPDCPLLAPLNDSRVVHAVALATFNAESNGSYLQLVEISR	0	0.21	0.16	0.37	1
VWPRRPTGEVYDIEIDTLETTCHVLDPTPLANCSVR	0	0.02	0.03	0.05	0
HTFSGVASVESSSGEAFHVGK	40.96	10.00	0.03	50.99	0
HTFSGVASVESSSGEAFHVGKTPIVGQPSIPGGPVR	1.16	2.50	0.00	3.66	1
KLCPDCPLLAPLNDSR	1.93	1.37	1.48	4.78	0
PTGEVYDIEIDTLETTCHVLDPTPLANCSVR	0.12	0.46	0.58	1.16	0
PTGEVYDIEIDTLETTCHVLDPTPLANCSVRQQTQHAVEGDCDIHVLK	0	0.01	0.01	0.02	1
QQTQHAVEGDCDIHVLK	10.44	5.20	0.01	15.65	0
RPTGEVYDIEIDTLETTCHVLDPTPLANCSVR	0.03	0.09	0.51	0.63	1
VVHAVEVALATFNAESNGSYLQLVEISR	0.03	0.25	0.15	0.43	0
VVHAVEVALATFNAESNGSYLQLVEISRAQFVPLPVSVSVEFAVAATDCIAK	0.03	0.14	0.15	0.32	1

The peptides containing either D or E are listed, in which they have been modified by ethanol or EDA. Proteins are immobilized on resin prior to derivatization. It is maximum to have one C-terminal esterification or amidation. One missed cleavage is included

### HILIC Enrichment of Derivatized Sialoglycopeptides

It is challenging to identify glycopeptides from global peptides without prior enrichment due to suppression by the abundant highly ionizable non-glycopeptides present in mixtures. Enrichment of glycopeptides can be performed using chemical immobilization or affinity chromatography [37]. Although chemical immobilization enrichment is effective for isolation of glycopeptides, oxidation of their glycans is required, which sacrifices the glycan moiety information [10]. Affinity chromatography utilizes hydrophilic interaction for selective enrichment of intact glycopeptides and, therefore, glycan specific information is retained [16, 38].

To facilitate enrichment by HILIC, we used EDA for carboxylic acid amidation instead of the aromatic pT that we have used previously in GIG applications [15, 34]. EDA has a chemical structure of H_2_N-CH_2_CH_2_-NH_2_ where one amine can react with carboxylic acid in the presence of EDC at pH 4–6. LC-MS experiments were analyzed by Byonic ® and analyzed by Byologic ™. Peptides were normalized by the total area of all peptides detected by LC-MS. HILIC enrichment without prior ethanol-EDA derivatization resulted in 32% of ion intensity present in identified ions and 0.095% of intensity in the flow-through; HILIC enrichment with prior ethanol-EDA modification resulted in 58% of ion intensity present in identified ions and 0.037% of intensity in the flow-through. These results demonstrate that ethanol-EDA derivatized glycopeptides can not only enriched by HILIC chromatography but also generate better coverage probably due to the better ionization, interaction with HILIC matrix, as well as stabilization of sialic acids.

### Identification of Linkages of Sialoglycopeptides

**Figure 4:**
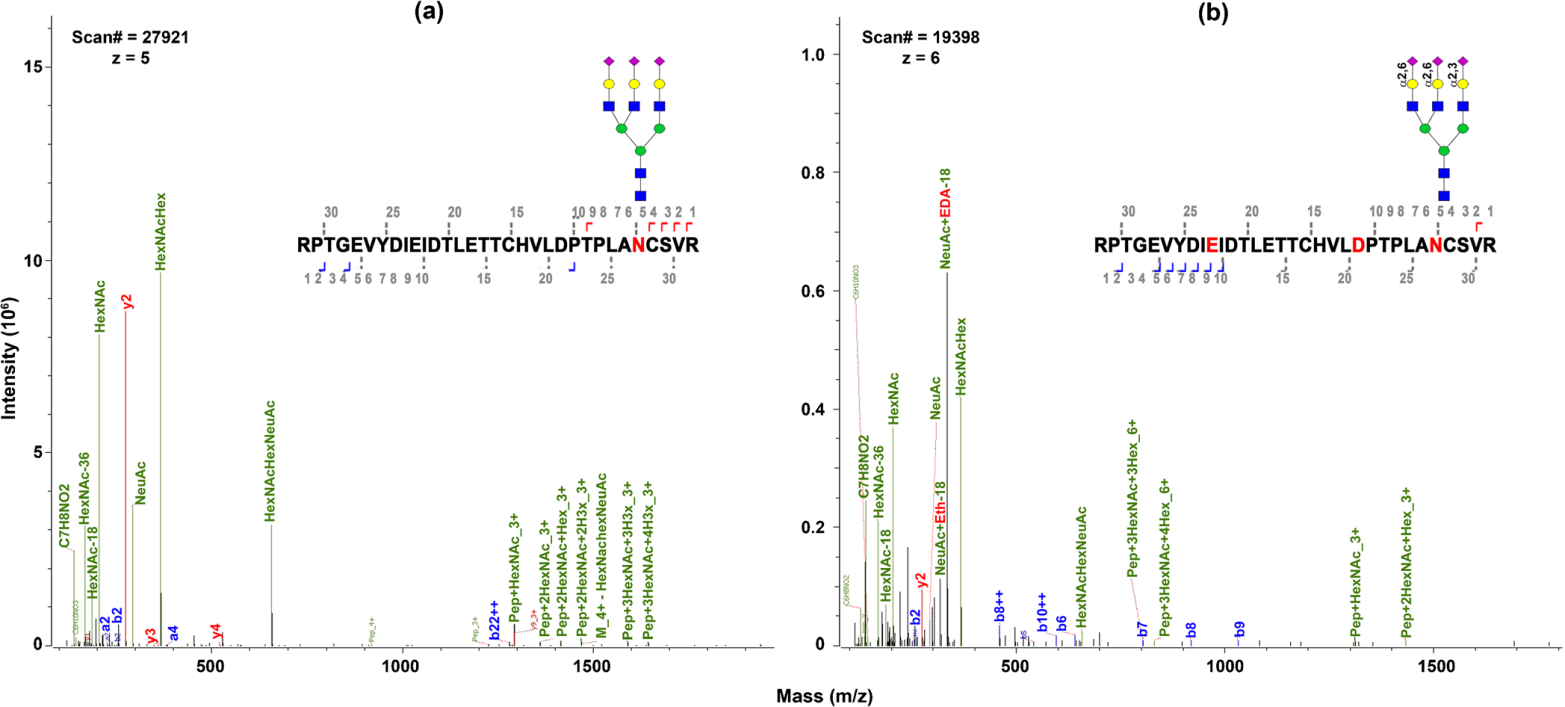
Fragment ions of sialoglycopeptides (**a**) without modification and (**b**) after ethanol-EDA derivatization. (**a**) Un-modified sialylated glycopeptide, RPTGVYDIEI-DTLETTCHVLDPTPLAN[(NeuAc)3Hex(6)HexNAc(5)]CSVR. (**b** Ethanol-EDA derivatized sialoglycopeptides. D and E were modified by ethyl esterification, while sialic acid α2,3-linked is labeled by EDA and α2,6-linked by ethanol

Ethanol-EDA modification on sialoglycopeptides results in identification of sialic acid linkages. Our previous work on ethanol-EDA modification of sialylated glycans showed that α2,6-linked and α2,3-linked sialic acids are labeled with different mass tags. As given in Table 1, one α2,6-linked sialic acid adds 28.0 Da and 2,3-linked sialic acid adds 42.0 Da after derivatization. Sialylated N-glycans were abundantly present in bovine fetuin studied here [39]. Figure 4 compares tri-antennary sialic acid (A3G3S3 or S3H6N5) on glycopeptide RPRGEVYDIEIDTLETTCHVLDPTPLA*NCS*VR. Without modification, we observed several oxonium ions (HexNAc, NeuAc, HexNAcHex, and HexNAcHexNeuAc) and Pep + HexNAc (Figure 4a). Daughter ions of native sialic acid of S3H6N5 (Figure 4a) show much higher intensity than for ethanol-EDA derivatized ones (Figure 4b) since daughter ion intensity generated from derivatized glycopeptides is divided among the unique masses imparted by the differential tags. Sialoglycopeptide. In contrast, the derivatized glycopeptides have a few unique features: (a) more oxonium ions were detected, including sialic acid modified by ethanol and EDA; (b) linkages were determined by mass tag; (c) higher intensity on EDA-modified NeuAc was seen compared to un-modified NeuAc; (d) a higher mass charge, *z*, was observed since EDA carries additional amine after reaction (which forms ▬NH_3_^+^). The higher net positive charges may be attributed to the reduction in the number of carboxylic acid groups through EDA amidation [40].

**Figure 5:**
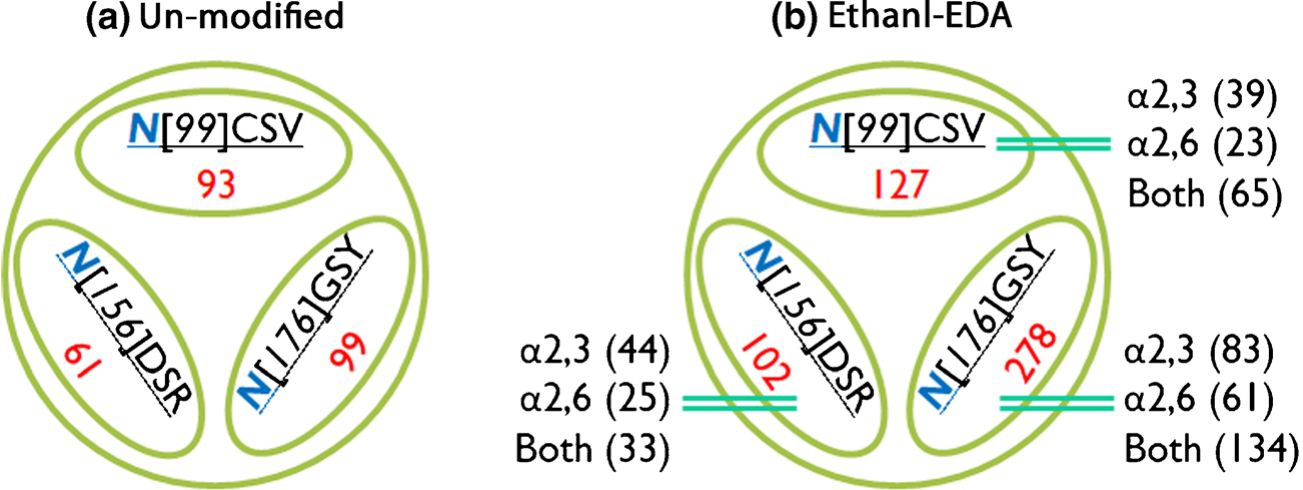
Identification of glycosites and glycopeptides from bovine fetuin with and without ethanol-EDA derivatization. (**a**) Three glycosites *N*[99]CSV, *N*[156]DSR, and *N*[176]GSY, containing 93, 61, 99 unique sialoglycopeptides, respectively (in triplicates), on native fetuin after HILIC enrichment. (**b**) Three glycosites containing 127, 102, and 278 sialoglycopeptides after ethanol-EDA modification. Sialic acid linkage is differentially determined by IntactGIG-HILIC

We compared unique sialoglycopeptides from un-modified and ethanol-EDA modified bovine fetuin. Several conclusions have been made based on results illustrated in Figure 5. First, all three glycosites are identified with or without derivatization, including N-99, N-156, and N-176 [41]. This demonstrates that on-resin protein digestion is robust for analysis of glycopeptides. The un-modified method only provides overall identification of sialoglycopeptides without information about their linkage. On the other hand, ethanol-EDA (Figure 5b) pinpoints the linkages of each sialylated glycopeptide. Importantly, more sialoglycopeptides are observed after ethanol-EDA modification. For example, glycan S3H6N5 contains different isomers, such as S3(2,6)H6N5, S2(2,3)S1(2,6)H6N5, S3(2,3)H6N5, and S1(2,3)S2(2,6)H6N5 [42, 43]. The same glycopeptide can be detected as four unique linkage-specific species after derivatization, revealing complexity at the site of glycosylation for sialoglycopeptides.

### Sialoglycoproteins in Human Serum

**Figure 6:**
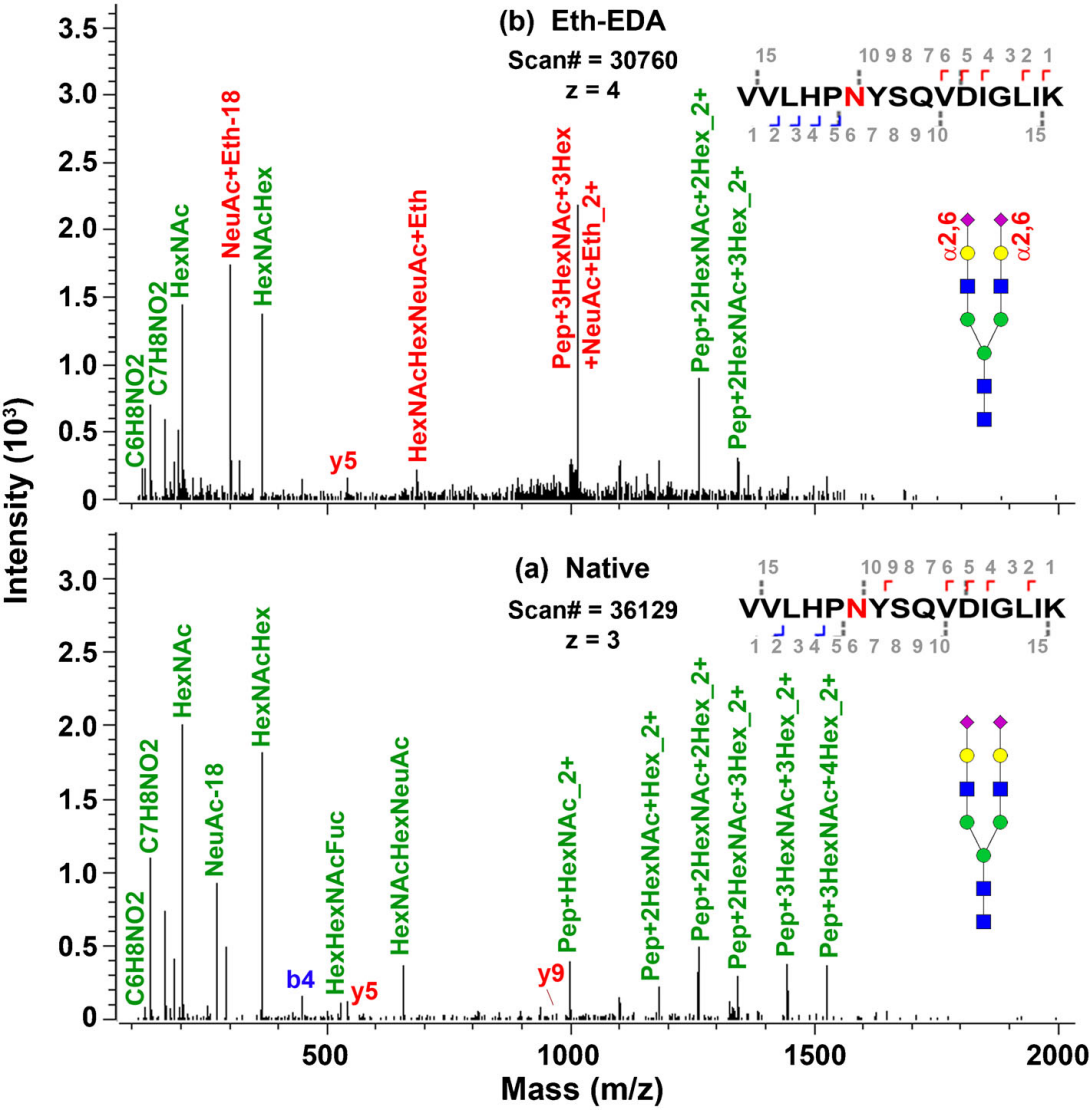
MS/MS spectra of one sialoglycopeptide identified from human serum haptoglobin. (**a**) Native sialoglycopeptides without derivatization. (**b**) Eth-EDA derivatized α2,6-linked sialoglycopeptide, VVLHP***N***[S2(2,6)H5N4]YSQVDIGLIK

We further applied IntactGIG method for analysis of human serum sialoglycopeptides. Many serum glycoproteins are highly sialylated and their most abundant glycans are S2H5N2 [11, 17]. We examined several glycoproteins, including alpha 1-acid glycoprotein, alpha 2-macroglobulin, amyloid-like protein 1, cadherin-2, endoplasmin, haptoglobin, hemopexin, integrin alpha-2, integrin beta-1, and titin [44, 45]. As shown in Table 4, most are sialylated glycoproteins, e.g., alpha-2-macroglobulin consists of S2H5N4, S2H5N4F1, and S1H5N4. Among those sialoglycopeptides, α2,6-linked sialic acids are associated with their glycopeptides. The spectra for this glycopeptides with (top) and without derivatization (bottom) are shown in Figure 6. Clearly shown is sialyl ethyl esterification, such as NeuAc + Eth and HexNAcHexNeuAc + Eth. Endoplasmin has α2,3-linked sialic acids on glycopeptides LGVIEDHS***N***[S2(2,3)H5N4]RTR. On the other hand, haptoglobin has both linked sialic acids on its glycopeptide, S1(2,6)S1(2,3)H5N4; similarly, glycopeptide ANKT has both linked sialic acids [S1(2,6)S1(2,3)H5N4F1. The differentiation of those linkages is important for studying the role of linkage in human diseases. This approach may be applied for sialoglycoprotein biomarker discovery in in human serum or other applications where the biology indicates functional differences dictated by sialyl linkage.

**Table 4:** Identification of Sialoglycopeptides from Human Serum Using IntactGIG Method. α2,6-Linked Sialoglycopeptides Are Abundantly Present in Human Serum

Glycoprotein	Uniprot ID	Glycosite	Sequence	Mass tag on glycan	Glycan
Alpha 1-acid glycoprotein	AAB33887	56	R.NeEYnK.S	HexNAc(3)Hex(4)	H4N3
	AAB33887	103	R.EnGTISR.Y	HexNAc(2)	N2
	AAB33887	33	M.ALSWVLTVLSLLPLLEAQIPLCANLVPVPITnATLDQITGK.W	HexNAc(2)	N2
Alpha-2-macroglobulin	P01023	70	R.GnR.S	HexNAc(3)Hex(4)	H4N3
				HexNAc(4)Hex(5)NeuAc(2) 56.062582	S2(2,6)H5N4
				HexNAc(4)Hex(5)Fuc(1)NeuAc(2)Na(2) 56.06258067	S2(2,6)H5N4F1 [2Na]
				HexNAc(4)Hex(5)NeuAc(1) 28.031275	S1H5N4
				HexNAc(4)Hex(5)Fuc(1)NeuAc(2) 56.062574	S2(2,6)H5N4F1
				HexNAc(2)Hex(7)	H7N2
				HexNAc(4)Hex(4)	H4N4
Amyloid-like protein 1	P51693	461	R.FQVHTHLQVIEERVnQSLGLLDQNPHLAQELRPQIQELLHSEHLGPSELEAPAPGGSSEDK.G	HexNAc(4)Hex(4)NeuAc(1)Na(1) 42.05816434	S1(2,3)H4N4[Na]
Cadherin-2	P19022	338	R.IVSQAPSTPSPNMFTINnETGdIITVAAGLdREK.V	HexNAc(2)Fuc(1)	H2F1
Endoplasmin	P14625	502	K.LGVIEDHSnRTR.L	HexNAc(4)Hex(5)NeuAc(2) 84.116346	S2(2,3)H5N4
Haptoglobin	4WJG	150	K.VVLHPnYSQVDIGLIK.L	HexNAc(4)Hex(5)NeuAc(2) 56.062582	S2(2,6)H5N4
				HexNAc(4)Hex(5)NeuAc(2)Na(2) 70.08947067	S1(2,6)S1(2,3)H5N4[2Na]
Hemopexin	P02790	453	K.ALPQPQnVTSLLGCTH.-	HexNAc(4)Hex(5)NeuAc(2) 56.062582	S2(2,6)H5N4
		187	R.SWPAVGnCSSALR.W	HexNAc(4)Hex(5)NeuAc(2) 56.062582	S2(2,6)H5N4
Integrin alpha-2	P17301	1074	K.GEYFVnVTTR.I	HexNAc(2)Hex(3)	H3N2
Integrin beta-1	P05556	94	K.NKnVTNR.S	HexNAc(4)Hex(5)Fuc(1)NeuAc(1) 28.031267	S1(2,6)H5N4F1
Titin	Q8WZ42	21153	R.AnK.T	HexNAc(4)Hex(5)NeuAc(2) 56.062582	S2(2,6)H5N4
			R.AnK.T	HexNAc(4)Hex(5)Fuc(1)NeuAc(2)Na(2) 70.08946267	S1(2,6)S1(2,3)H5N4F1[2Na]

N, hexNAc; H, hexose; F, fucose; S, NeuAc

## Conclusions

It is challenging to accurately detect sialoglycopeptides using LC-ESI-MS/MS due to their fragility and ionization characteristics. Preservation of their structure throughout sample processing and LC-MS analysis can be achieved by sialic acid chemical stabilization. Here, we have developed methodology that not only stabilizes sialic acids present on glycopeptides but also adds mass tags to differentiate between the α2,3- and α2,6 linkage forms, which are commonly found to differentially function in biological systems.

In this study, we modify sialic acids using two-step derivatization via a chemoenzymatic solid-phase method. Sialic acid α2,6 linkages are first labeled by ethyl esterification. Next the α2,3 linkages are derivatized by EDA amidation. Our results revealed that amino acids D and E are pre-dominantly modified by ethyl esterification, with lesser degree modification by EDA amidation. The entire process is performed on solid-phase, thus excess amount of reagent can be used and subsequently removed by washing steps. By selecting non-aromatic reagents (or aliphatic molecule), the derivatized glycopeptides possess hydrophilic characteristics. Thus, they can be enriched by HILIC-SPE chromatography. Our method provides several unique features for analysis of intact glycopeptides: (1) the aliphatic reagent (EDA) prevails hydrophilicity of the derivatized sialoglycopeptides, enabling sialoglycopeptide enrichment by HILIC chromatography; (2) the free amine on EDA brings additional charge for better electrospray ionization; and (3) differential sialic acid linkages of sialoglycopeptides by mass tags. This approach is useful for identification of linkage-specific glycosylation for better understanding of how linkages affect biological processes.
